# Smooth muscle degeneration of the mesenteric and branching veins causing ischemic enteritis: a case report

**DOI:** 10.1186/s40792-021-01353-x

**Published:** 2022-01-04

**Authors:** Taiki Sunakawa, Nobuo Ito, Ryo Moriyasu, Nobuya Seki, Daisuke Takeuchi, Kotaro Sasahara

**Affiliations:** 1 Department of Surgery, Aizawa Hospital, 2-5-1Honjo, Matsumoto, Japan; 2 Department of Pathology, Aizawa Hospital, 2-5-1Honjo, Matsumoto, Japan

**Keywords:** Intestinal ischemia, Ischemic enteritis, Small bowel obstruction, Smooth muscle degeneration, Idiopathic mesenteric phlebosclerosis, Idiopathic myointimal hyperplasia of mesenteric veins

## Abstract

**Background:**

Ischemic bowel injuries are generally caused by arteriosclerosis, thromboembolism, or vasculitis. Ischemic enteritis is less common than ischemic colitis because of the rich collateral arteries of the small intestine. In the present case, smooth muscle degeneration of the mesenteric to the submucosal veins caused ischemic enteritis and small bowel obstruction.

**Case presentation:**

An 85-year-old woman with recurrent enteritis eventually developed small bowel obstruction. We performed laparoscopic partial resection of the small intestine. The pathological findings revealed smooth muscle degeneration of the mesenteric veins that caused ischemic enteritis. Venous changes were detected not only in the injured region, but also in a part of the normal region of the resected specimen. She continued to experience some minor symptoms postoperatively; however, these symptoms subsided in a short period with medicine discontinuation.

**Conclusion:**

This report shows the possibility that a disease causes ischemic enteritis with unique venous pathological changes and may recur postoperatively.

## Background

Intestinal ischemia typically develops because of arterial thromboembolism. Other common causes of ischemic bowel disorders are venous thrombosis or vasculitis [1–3]. Ischemic enteritis is more uncommon than ischemic colitis because of the rich blood flow in the small bowel [4, 5]. Surgery is often required for diagnosis and treatment, owing to the severity of the stricture and the difficulty of examination using endoscopy [6, 7]. We present the case of a patient who exhibited rare pathological changes of the mesentery to branching veins, causing ischemic enteritis associated with small bowel obstruction.

## Case presentation

**Fig. 1:**
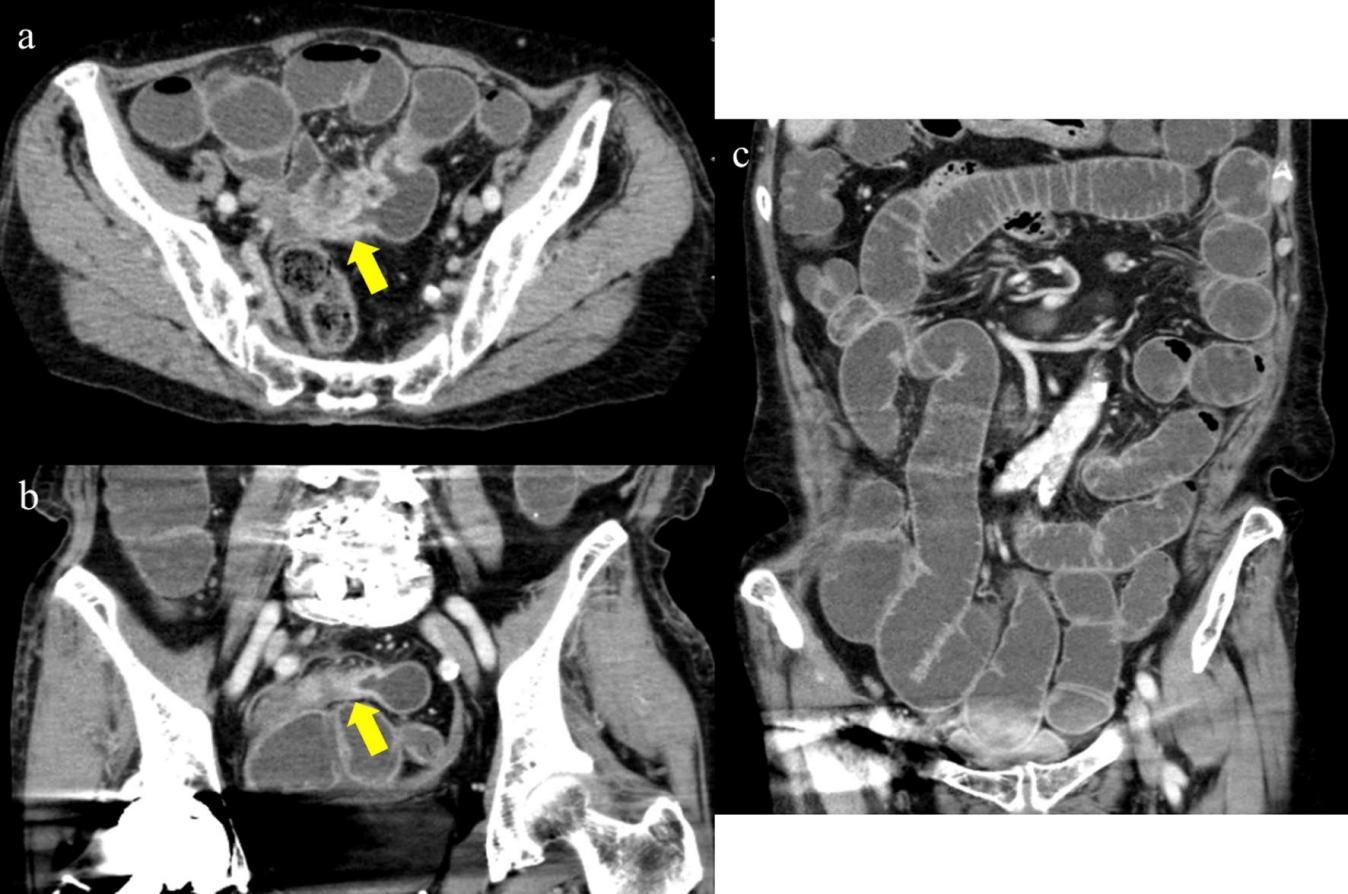
Abdominal CT findings. CT images revealed wall thickening and stricture of the small bowel (arrow) and fat stranding around the lesion (**a** axial view, **b** coronal view). A wide range of small bowel distension and fluid accumulation in the lumen were observed, showing small bowel obstruction (**c**). There were no specific findings indicating disorder of blood circulation or any characteristics, such as calcification over the mesenteric veins and the bowel walls

**Fig. 2:**
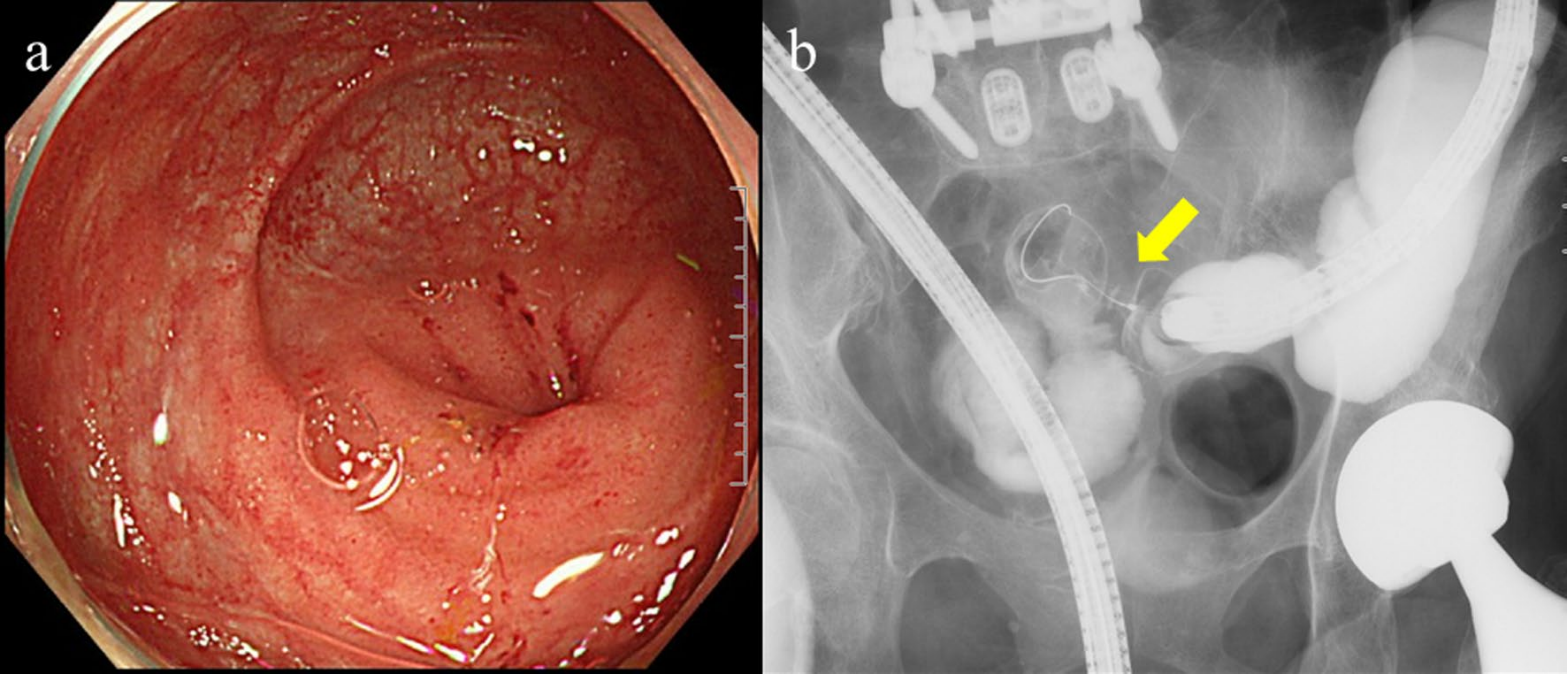
Colonoscopy and contrast study findings. Colonoscopy showed circumferential stenosis of the terminal ileum and a little edematous red mucosa, but no obvious epithelial lesion (**a**). Contrast study demonstrated the narrowed section of the ileum (arrow) (**b**)

**Fig. 3:**
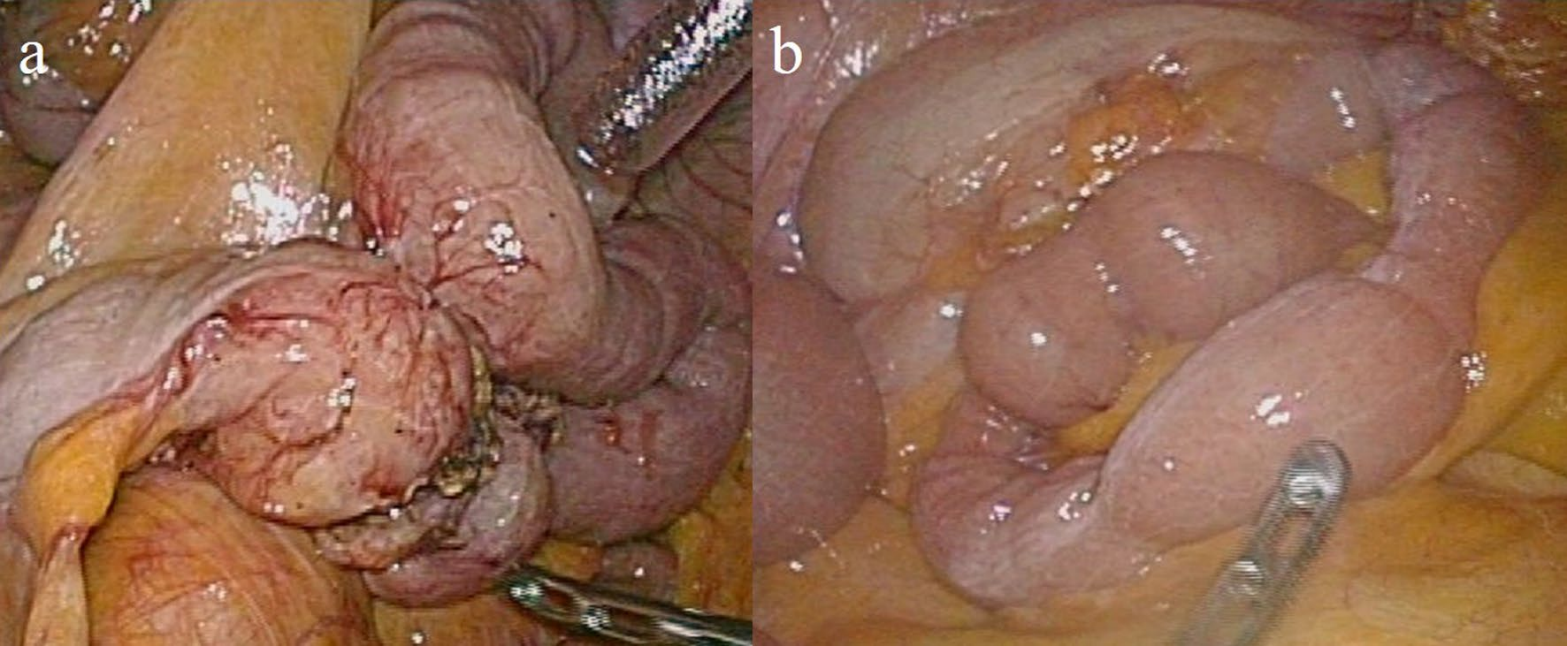
Intraoperative findings. Stenosis part of the ileum showed constriction of the serosa, without any obvious findings of ischemia (**a**). There were no inflammatory or ischemic lesions in the remaining part of the small bowel, mesentery, and intra-abdominal fat (**b**)

**Fig. 4:**
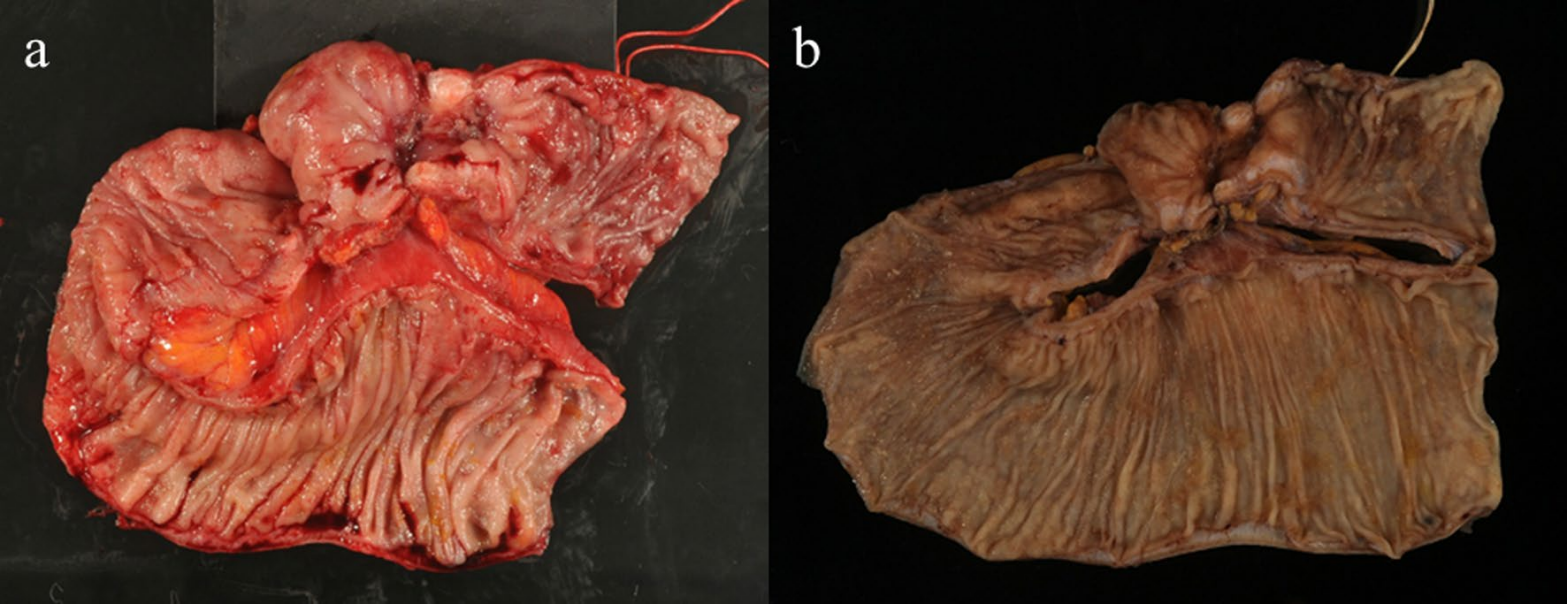
Gross specimen. Gross appearance revealed deep ulceration that penetrates the mesentery and caused stenosis of the ileum and the dilated proximal bowel (**a** fresh, **b** after formalin fixative)

**Fig. 5:**
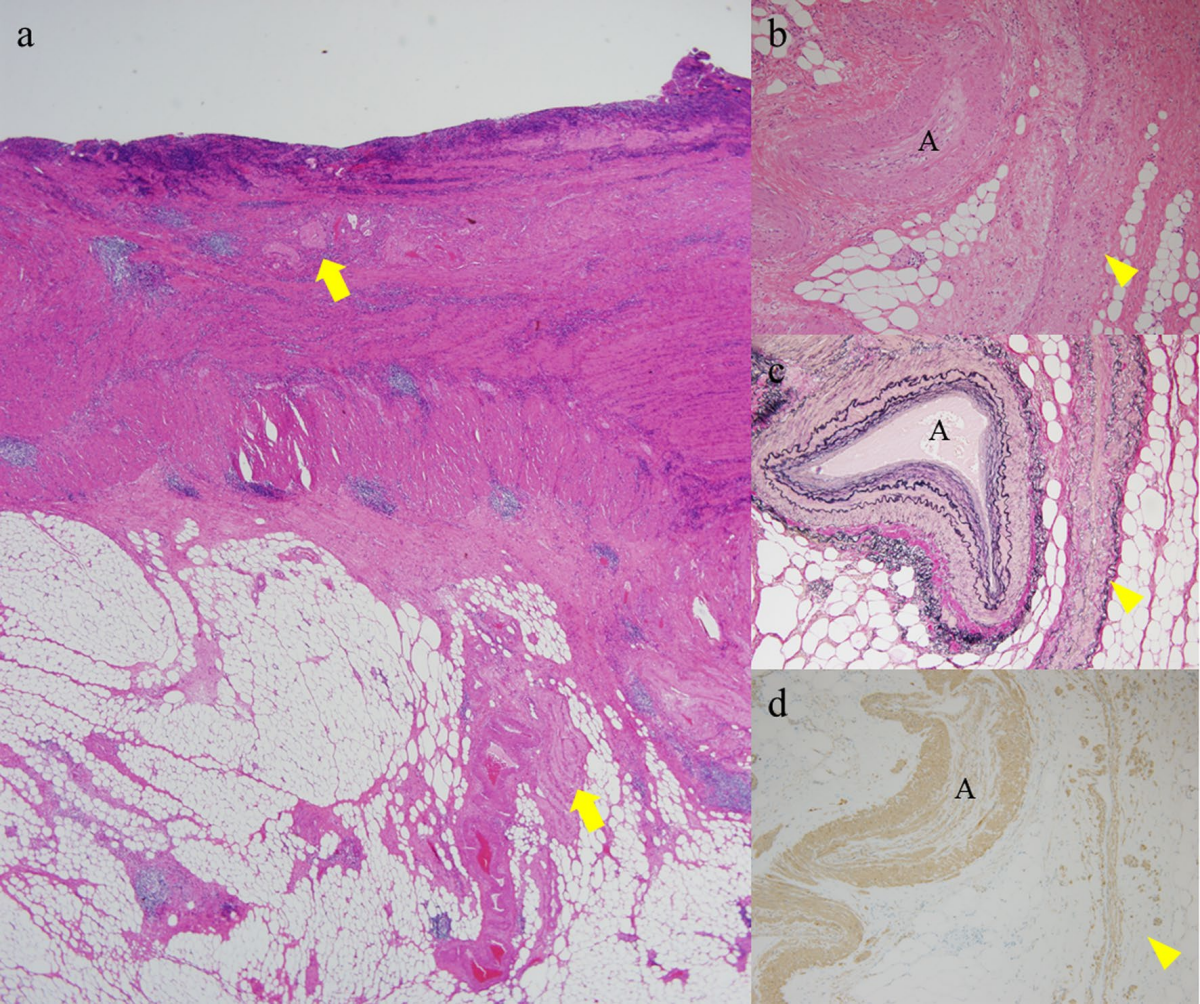
Histopathological findings of the ischemic lesion of the small bowel. Venous wall from the mesentery to the submucosa showed degeneration (arrows) that caused ulceration and some other ischemic changes in the affected mucosa (**a** HE, × 20). Mesenteric vein showed degeneration, decrease in the number of smooth muscle cells, and narrowing of the lumen (arrowhead). The accompanying artery was patent; however, mild sclerotic changes were observed (A) (**b** HE, × 100 **c** EvG, × 100 **d** smooth muscle actin immunostaining, × 100)

**Fig. 6:**
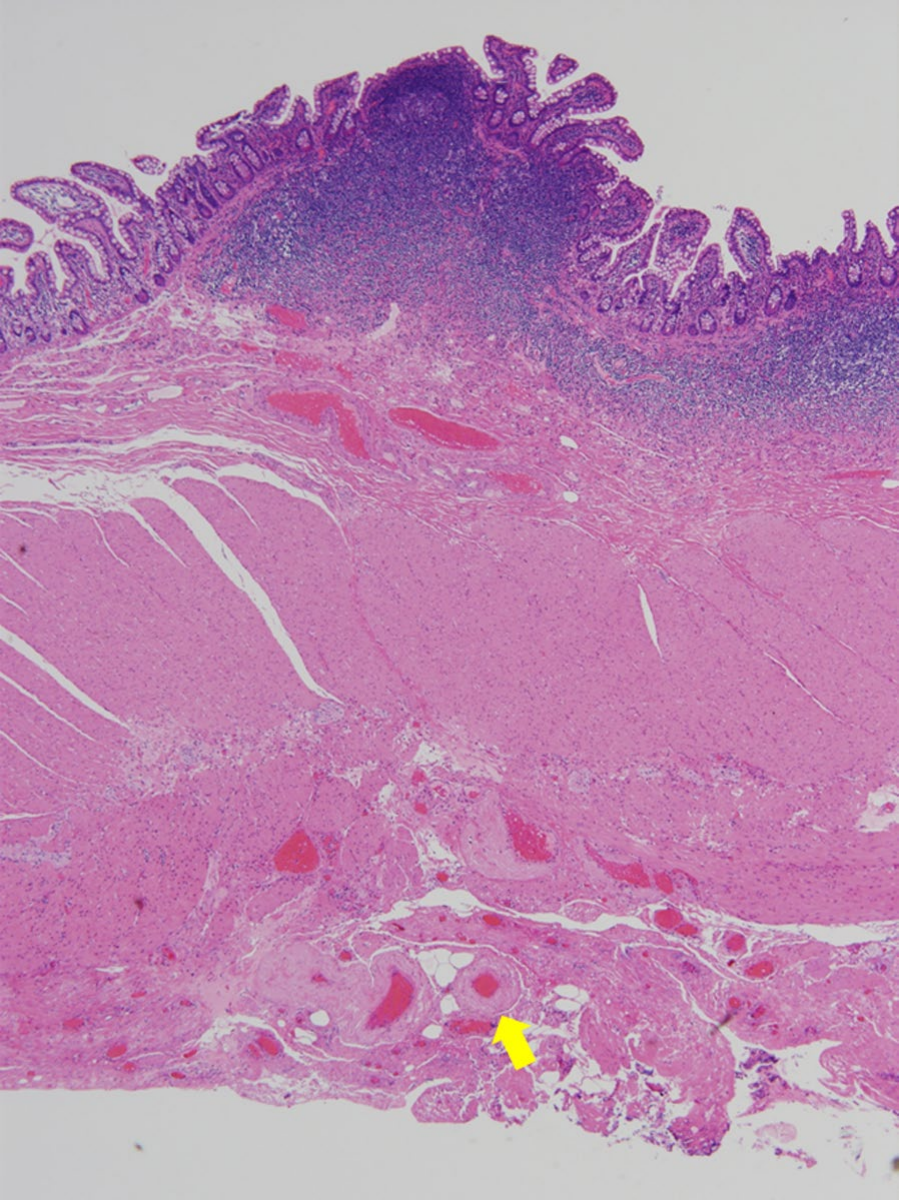
Pathological findings of the no stenotic part of the resected bowel. The venous wall of the mesentery showed degeneration of the smooth muscle cells and thickening; however, the lumen was patent (arrow) (HE, × 20). Mild ischemic mucosal changes were detected, such as low, flat, and wide villi

An 85-year-old woman was referred to our hospital because of nausea, vomiting, and right lower abdominal pain. She had been admitted to our hospital 2 months previously because of infection enteritis. After her hospital discharge, she had experienced diarrhea and constipation repeatedly. Her medical history included appendicitis, collagenous colitis, myocardial infarction, hypertension, and asthma. Her regular medications included aspirin, several depressors, and stomach medication; she was not taking any herbal medication. On examination, her body temperature was 38.5 °C, and she had shaking chill and tenderness in the right abdomen. Her blood workup showed a white blood cell count of 20,480/μL; the levels of all other blood parameters, including C-reactive protein, were normal. An abdominal computed tomography (CT) revealed thickened wall and stricture of the ileum near the terminal ileum, with dilation and fluid accumulation in the small bowel (Fig. 1). No decreased bowel wall enhancement was detected and there were some enlarged lymph nodes in the surrounding mesentery. She was diagnosed with recurrent infection enteritis associated with ileus and was admitted to the hospital for bowel rest and antibiotic treatment. Although she showed improvement soon after hospitalization, her symptoms eventually relapsed on day 9. Colonoscopy showed circumferential stenosis of the ileum 20 cm from the ileocecal valve, with no epithelial lesion (Fig. 2). Enterography revealed about 2 cm circumferential stenosis. As neoplastic tumorous lesion or scar formation after inflammation was suspected, we performed laparoscopic partial small bowel resection. Intraoperative findings showed a depressed part of the ileum that corresponded to the stricture lesion; however, no other inflammatory lesions were observed in the small bowel and the mesentery, and no ascites was noted (Fig. 3). The stenotic part of the ileum was located 30 cm from the ileocecal valve, and we performed partial bowel resection, saving the ileocecal valve. Gross findings showed ulceration and distention of the proximal small bowel (Fig. 4). Pathological evaluation showed ulcer formation, fibrotic change of the lamina propria and submucosa, crypt destruction, and some other changes induced by ischemia. These ischemic lesions were caused by smooth muscle degeneration of the venous wall from the mesentery to the submucosa (Fig. 5). No calcification and hyalinization were observed in the venous walls. Congo red staining did not demonstrate amyloid deposition. In contrast, the arteries accompanying these veins were all intact; however, mild sclerotic changes were detected. Furthermore, such venous pathological changes are found in an area with slight mucosal changes (Fig. 6), which was located at the proximal end of the specimen. We diagnosed the patient with ischemic enteritis that resulted from smooth muscle degeneration of the mesenteric and submucosal veins, associated with bowel obstruction by postinflammatory fibrosis. She was discharged after the treatment for surgical site infection. About 1.5 years postoperatively, she was referred to our hospital again because of chronic diarrhea. Colonoscopy and biopsy were conducted, and she was diagnosed with collagenous colitis with suspected eosinophilic colitis. She has been followed up without treatment because the adjustments to her medication successfully alleviated her symptoms.

## Discussion

Intestinal ischemia is mainly caused by arteriosclerosis and arterial thromboembolism. [1–3]. Ischemic enteritis is more uncommon than ischemic colitis because of the rich blood flow in the small intestine [4, 5]. In this case, we identified two interesting pathological characteristics that cause ischemic enteritis. First, smooth muscle degeneration occurred only in the veins of the mesentery to the submucosa, leading to intestinal ischemia. Second, those changes were detected not only in the part of the stenotic lesion, but also in the normal part of the small bowel.

In general, arteriosclerosis and thromboembolism are the main causes of intestinal ischemia. Venous occlusion is an uncommon cause of intestinal ischemia and is mainly caused by venous thrombosis or vasculitis associated with conditions, such as systemic lupus erythematosus, and Behçet disease [1–3]. In this case, smooth muscle degeneration of the venous wall from the mesentery to the submucosa caused intestinal ischemic changes, resulting in luminal narrowing by fibrosis. Immunohistochemical examination after Elastica-van Gieson and smooth muscle actin staining can reveal the degeneration of the smooth muscles in the mesenteric veins. To the best our knowledge, smooth muscle degeneration in the mesenteric and branching veins was not reported as the cause of intestinal ischemia. Moreover, such pathological changes were detected only in the veins, not in the arteries. Similar to that in this case, idiopathic mesenteric phlebosclerosis (IMP) and idiopathic myointimal hyperplasia of mesenteric veins (IMHMV) are rare entities that selectively affect the veins and cause intestinal ischemia [1–3]. However, in the reported cases, these two diseases usually cause ischemic colitis and exhibit some distinct clinicopathological features. With respect to the pathological findings, IMP is characterized by sclerosis of the mesenteric vein and its branches, as well as fibrosis, hyaline degeneration, calcification and thickening of the colon wall [8]. Special staining techniques such as Elastica-van Gieson and Azan-Mallory staining can detect these pathological changes. On the other hand, IMHMV has venous stricture or occlusion caused by myointimal hyperplasia without any vasculitis or thrombosis [9, 10]. Elastin stains can detect that internal elastic laminae are absent from the veins. IMP, IMHMV, and this presented case cause chronic intestinal ischemia via venous stricture, occlusion, or stasis congestion; however, each of them reveals unique pathological changes of the mesenteric veins.

Furthermore, the pathological changes in the veins were detected not only in the part of the ischemic lesion, but also in the normal part of the small bowel. Although mild venous changes were detected in parts without stenosis, we think that severe anomalous venous drainage did not occur, and the mucosa was not severely damaged. The continuity of the venous changes between the stenotic and the normal parts was uncertain. In a previous report, surgical resection was mostly required for ischemic enteritis with stenosis, and postoperative recurrence ratio of ischemic enteritis is uncertain [4, 11, 12]. Our patient also underwent surgery, and exhibited smooth muscle changes of the veins in the part without ischemic mucosal damage and continued to experience minor symptoms postoperatively. Although she was diagnosed with collagenous colitis and suspected to have eosinophilic colitis, the venous lesions may have contributed to the symptoms. Hence, we might need to consider this disease as the cause when small bowel stenosis recurs.

The differential diagnosis for small bowel stricture includes inflammatory bowel disorders, malignant tumor, tuberculosis, nonsteroidal anti-inflammatory drug-associated enteritis, trauma, antiphospholipid antibody syndrome, and abdominal adhesions related to previous surgery [4, 6, 7, 13]. In addition, intestinal anisakiasis can exhibit a similar clinical course, making it important to take a history of the illness [7].

Etiology for this presented disease is unclear. In many cases of elderly patients, small bowel stenosis is caused by neoplastic lesion such as carcinomas and lymphomas. Ischemic enteritis, rheumatoid vasculitis, and diaphragm disease have been reported as the rare causes of small bowel stenosis [4, 14–16]. In previous reports, most elderly patients with stenotic ischemic enteritis have underlying diseases including hypertension, diabetes mellitus, atrial fibrillation, cerebral infarction, and ischemic heart disease [4, 14]. The case presented in this study had a medical history of ischemic heart disease and hypertension, thereby suggesting that ischemic enteritis is more common in elderly patients with underlying cardiovascular disease.

Generally, intestinal stenosis or obstruction cases with severe symptoms undergo surgical intervention. With regard to ischemic enteritis, two types have been described on the basis of the cause: occlusive and nonocclusive [13, 17]. In both types, ischemic enteritis can lead to fatal conditions with intestinal necrosis and peritonitis. Therefore, early surgical intervention or endovascular therapy is generally required. If the symptoms are not severe and resolved by conservative therapy, further intervention is not indispensable. Furthermore, balloon dilation with double-balloon endoscopy is effective in treating the stricture and preventing the need for surgery in some patients [6]. In particular, when the stricture length is < 3 cm, balloon dilation can be more effective. Our patient exhibited a stricture length of approximately 2 cm. However, the preoperative diagnosis was tumorous lesion; therefore, surgery was performed.

With respect to future follow-ups, detection of small bowel disorders is challenging. Previous reports have shown that the recent development of double-balloon endoscopy or capsule endoscopy has enabled the observation and detection of small bowel lesions [6, 7, 18]. Double-balloon endoscopy or capsule endoscopy may be required and it is important to include ischemic enteritis caused by this disease in the differential diagnosis when the patient experiences a relapse of abdominal symptoms. It is crucial to make the correct decision regarding the appropriate examination and treatment, depending on the symptom severity and differential diagnosis.

## Conclusions

We present the case of a patient with ischemic enteritis caused by rare mesenteric venous changes. This disease could lead to ischemic enteritis without arteriosclerosis, thromboembolism or vasculitis. In addition, the uncommon pathological changes were also detected in the intact part of small bowel; therefore, ischemic enteritis and small bowel stenosis may recur after surgery because of this pathological abnormality.

## Data Availability

The dataset supporting the conclusions of this article is included within the article.

## Abbreviations

IMP: Idiopathic mesenteric phlebosclerosis
IMHMV: Idiopathic myointimal hyperplasia of mesenteric veins
CT: Computed tomography
